# Supplementary material for: A UMLS-based spell checker for natural language processing in vaccine safety
Source: BMC Med Inform Decis Mak. 2007 Feb 12;7:3. doi: 10.1186/1472-6947-7-3 (PMC1805499; doi:10.1186/1472-6947-7-3)
Supplement: Additional file 13 — RAP application directory. Sets up directory for RAP (RDF application for PHP) source code [file 1472-6947-7-3-S13.gz › rap/doc/phpdoc/classtrees_default.html]

xml version="1.0" encoding="iso-8859-1"?


## Root class ATOM

- ATOM

## Root class ATOM\_RES

- ATOM\_RES

## Root class DC

- DC

## Root class DC\_RES

- DC\_RES

## Root class FOAF

- FOAF

## Root class FOAF\_RES

- FOAF\_RES

## Root class OWL

- OWL

## Root class OWL\_RES

- OWL\_RES

## Root class RDF

- RDF

## Root class RDFS

- RDFS

## Root class RDFS\_RES

- RDFS\_RES

## Root class RDF\_RES

- RDF\_RES

## Root class RSS

- RSS

## Root class RSS\_RES

- RSS\_RES

## Root class VCARD

- VCARD

## Root class VCARD\_RES

- VCARD\_RES

Documentation generated on Fri, 17 Dec 2004 16:14:20 +0100 by phpDocumentor 1.3.0RC3
